# Does diagnostic testing always decrease antibiotics prescriptions?

**DOI:** 10.1007/s10198-022-01488-0

**Published:** 2022-08-03

**Authors:** F. Antoñanzas, C. A. Juárez-Castelló, R. Rodríguez-Ibeas

**Affiliations:** Department of Economics, University of La Rioja, La Cigüeña 60, 26006 Logrono, Spain

**Keywords:** Antibiotics, Prescriptions, Diagnostic tests, Infectious diseases, Antimicrobial resistances, I11, I12

## Abstract

**Background:**

Empiric prescription to treat infectious diseases in community care settings has caused antibiotics to be overprescribed, increasing antimicrobial resistance (AMR). To reduce antibiotics prescription, the use of point-of-care diagnostic testing (POCT) has been suggested.

**Methods:**

We present a stylized static theoretical economic model to analyse whether the use of POCT always decreases antibiotics prescriptions. We consider the interaction of a group of doctors who differ in their level of concern about AMR when prescribing with a firm selling a POCT, and we characterize the price set by the manufacturer and doctors’ decision to employ POCT.

**Results:**

We found that the number of antibiotics prescriptions is not always lower. This result depends on the distribution of the doctors’ concern about AMR as there is a proportion of doctors who use POCT and then prescribe antibiotics while other doctors change their prescribing behaviour after using POCT and stop giving antibiotics to patients who do not benefit from them. When the proportion of patients who need antibiotic treatment is higher than the proportion of doctors who use POCT and stop prescribing unnecessary antibiotics, the number of antibiotics prescriptions is larger. Our analysis also shows that the use of POCT improves health outcomes.

**Conclusions:**

We should be very careful when we assert that POCT reduces antibiotics prescriptions as there are situations in which the opposite effect occurs.

## Introduction

Empiric prescription to treat infectious diseases in community care settings has caused antibiotics to be overprescribed. It is considered, for example, that only 10% of patients with an acute cough who seek medical attention should be treated with antibiotics, whereas the actual proportion of antibiotics prescribed in EU countries is reported to be 50% overall with a range of between 20 and 80% [1, 2]. The excessive use of antibiotics has favoured the development of antimicrobial resistance (AMR), which makes antibiotics less effective. The World Health Organization (WHO) considers this a public health problem. Antibiotics should be prescribed only when patients need them, and their prescription otherwise should be avoided. However, doctors face uncertainty when they diagnose and treat infectious diseases, and frequently prescribe antibiotics, even when they are not effective. In general, antibiotics are not effective to treat respiratory infections of viral origin. In addition, there may be the inappropriate empirical antibiotic treatment if the antibiotic shows no effect against the isolated microorganism.

Likewise, few doctors consider the costs of AMR when prescribing. It has been argued that the use of point-of-care diagnostic testing (POCT) may guide antibiotics prescription [3]. It may reduce the number of antibiotic prescriptions in the short term, and help to reduce bacterial resistance in the medium and long term [4, 5]. In this article, we analyse whether the use of POCT always decreases antibiotics prescriptions.

We present a stylized static theoretical model to analyse, from an economic perspective, the interaction between a manufacturer of a POCT and a population of doctors concerned about AMR who have to decide whether to use POCT when treating their patients. As reported by Kaae et al*.* [6], we consider that doctors acknowledge AMR, and consider its risk in their daily prescribing practice. We characterize the price for the POCT set by the manufacturer and its impact on doctors’ decision to employ it. Our analysis shows that it should not be taken for granted that the availability of diagnostic testing necessarily reduces antibiotics prescriptions. Intuitively, the use of POCT makes some doctors prescribe antibiotics while others stop doing so. The final effect hinges upon the sizes of both groups of doctors. The paper is structured as follows. In the next section, we describe the model. “The prescription” characterizes empiric prescription and prescription when a POCT is available. In “The determination of the price of the diagnostic test”, we determine the price for the POCT chosen by the manufacturer, and in “Antibiotics prescriptions and health outcomes” we analyse how the number of antibiotics prescriptions changes due to the use of POCT. We close the article with a discussion of our results and some conclusions.

## The model

We consider a population of doctors, whose size is normalized to $1$, with each doctor attending in his/her primary care facility a population of patients of size 1 suffering from an infectious disease (for example, a respiratory tract infection) that can be treated with antibiotics. Within each population of patients, we assume that antibiotics are effective only for a proportion $g\in \left({0,1}\right)$ of patients but doctors are unable to identify these patients. Thus, doctors cannot prescribe selectively and give antibiotics only to those patients for whom they are effective. The proportion $g$ is common knowledge.^1^

Doctors differ in their concern about future AMR due to antibiotics prescription. We measure doctors’ concern with a random variable $r$ distributed across the population of doctors according to a cumulative distribution function $F(r)$ on the domain $\left[{0,1}\right]$, with density function $f\left(r\right)>0.$ Let $c$ be the cost of the antimicrobial resistance associated with each unit of antibiotics given.^2^ Doctors prescribe either one unit of antibiotics or nothing. For a doctor with AMR concern $r$, the cost of AMR when he/she prescribes antibiotics is then $rc$.^3^ The patient receives benefit $B$ if the antibiotic is effective. $B$ is the economic value of the health gain (i.e. the price people are willing to pay for good health). When the patient is not treated or the antibiotics are not effective, the benefit is $\lambda B$, with $\lambda <1$. We implicitly assume that the patient gets better in the future regardless, although the benefit is lower. The price of the antibiotics $p\ge 0$ is exogenous. A manufacturer produces and markets a POCT. The POCT determines without error the patients for whom the antibiotics are effective. We assume, for simplicity, that the unitary production cost of the POCT is zero. Therefore, the profits for the firm are equal to the revenues from the sales of the POCT.

We model the interaction between the firm and the doctors as a two-stage game. In the first stage, the firm chooses the price $t\ge 0$ of the POCT to maximize its profits. In the second stage, each doctor, given the price $t$, chooses one of the two available strategies. Each doctor may choose not to use POCT and decide on the treatments empirically. Alternatively, the doctor may use POCT and treat the patients according to the results of the test. Each doctor chooses the strategy that maximizes the aggregated expected net health benefits, defined as the difference between the patients’ benefits and the treatment costs, including the costs of AMR, if any, and the cost of the POCT. We assume a perfect agency relationship between the doctors and the primary care facilities as well as between the doctors and the patients. Thus, doctors care about the health outcomes and all the treatment costs. We characterize the subgame perfect equilibrium of the game using backward induction; in particular, we find the optimal price $t$ chosen by the firm and the subset of doctors who use POCT.

## The prescription

### Empiric prescription

First, we analyse the empiric prescription when doctors do not use the diagnostic test. As doctors are not able to identify the patients for whom antibiotics are effective, they will give the same treatment to all (either antibiotics or nothing). If doctor $r$ prescribes antibiotics, the expected net benefit is $gB-p+\left(1-g\right)\lambda B-rc$. He/she prescribes the antibiotics to all patients, with the expected benefit being $gB$ $+\left(1-g\right)\lambda B$, while the treatment costs $p$ and the AMR $rc$.

If doctor $r$ does not prescribe antibiotics, the benefit is $\lambda B$. The degree of concern about AMR of the doctor who is indifferent between prescribing antibiotics or not satisfies:

$$gB - p + \left( {1 - g} \right)\lambda B - rc = \lambda B \Rightarrow \overline{r} = \frac{{g\left( {1 - \lambda } \right)B - p}}{c}$$

**Fig. 1:**
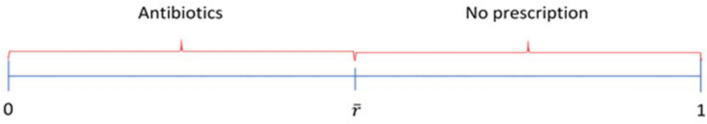
Empiric prescription

Doctors with $r>\overline{r}$ do not prescribe antibiotics. We assume $c>g\left(1-\lambda \right)B-p>0$, and therefore, $\overline{r}\in ({0,1})$ (Fig. 1).^4^

#### Proposition 1:

*When doctors do not use a diagnostic test to guide prescription, only those with* $r\le \overline{r}$ *prescribe antibiotics.*

### The prescription when POCT is used

Let us now assume that doctors can use a POCT that allows them to identify the patients for whom antibiotics are effective. If doctor $r$ use POCT, the expected net benefits are $g(B-p-rc$)$+\left(1-g\right)\lambda B-t$. Doctor $r$ prescribes antibiotics to the patients identified by POCT as responders. For these patients, the net benefit is $g(B-p-rc)$. Doctor $r$ leaves the remaining patients without treatment. For these patients, the benefit is $(1-g)\lambda B$. Finally, the POCT must be paid for.

The decision to use POCT depends on both the type of doctor and the price of the diagnostic test. Doctors with $r\le \overline{r }$ use the test if the expected net benefit is higher than the expected net benefit when they prescribe antibiotics to all patients (their decision when they did not use the diagnostic test):

$$\begin{gathered} g(B - p - rc) + \left( {1 - g} \right)\lambda B - t \ge gB - p + \left( {1 - g} \right)\lambda B - rc \Leftrightarrow t \le \left( {1 - g} \right)\left( {p + rc} \right) \hfill \\ \Updownarrow \hfill \\ r \ge \frac{{t - \left( {1 - g} \right)p}}{{\left( {1 - g} \right)c}} = r_l \left( t \right) \hfill \\ \end{gathered}$$

Therefore, no doctor with $r\le \overline{r }$ uses the diagnostic test unless ${r}_{l}(t)\le \overline{r}$. If we compare $\overline{r }$ y ${r}_{l}(t)$, we have:

$$\overline{r}{ \gtreqless }r_l \left( t \right) \Leftrightarrow g\left( {1 - g} \right)\left( {1 - \lambda } \right)B { \gtreqless }t$$

Thus, if $t> g\left(1-g\right)\left(1-\lambda \right)B$, it follows that ${r}_{l}\left(t\right)>\overline{r}$, and no doctor with $r\le \overline{r}$ uses the diagnostic test. If, on the contrary, $t\le g\left(1-g\right)\left(1-\lambda \right)B$, it follows that ${r}_{l}\left(t\right)\le \overline{r}$, and doctors with $r\in \left[ {r}_{l}(t),\overline{r}\right]$ use the diagnostic test.

Let us now focus on doctors with $r>\overline{r }$. They use the diagnostic test if the expected net benefit is higher than the expected net benefit when they do not prescribe antibiotics (their decision when they do not use the diagnostic test):

$$\begin{gathered} g(B - p - rR) + \left( {1 - g} \right)\lambda B - t \ge \lambda B \Leftrightarrow t \le g\left( {B\left( {1 - \lambda } \right) - p - rc} \right) \hfill \\ \Updownarrow \hfill \\ r \le \frac{{g\left( {B\left( {1 - \lambda } \right) - p} \right) - t}}{gc} = r_h \left( t \right) \hfill \\ \end{gathered}$$

Therefore, no doctor with $r>\overline{r }$ uses the diagnostic test unless ${r}_{h}(t)\ge \overline{r}$. If we compare $\overline{r}$ y ${r}_{h}(t)$, we have:

$$r_h \left( t \right) { \gtreqless }\overline{r} \Leftrightarrow g\left( {1 - g} \right)\left( {1 - \lambda } \right)B { \gtreqless }t$$

Thus, if $t> g\left(1-g\right)\left(1-\lambda \right)B$, it follows that ${r}_{h}\left(t\right)<\overline{r}$, and no doctor with $r>\overline{r}$ uses the diagnostic test. If, on the contrary, $t\le g\left(1-g\right)\left(1-\lambda \right)B$, it follows that ${r}_{h}\left(t\right)\ge \overline{r}$, and doctors with $r\in (\overline{r},{r}_{h}\left(t\right)]$ use the test. The proposition below summarizes the above analysis.

#### Proposition 2:

If $t\le g\left(1-g\right)\left(1-\lambda \right)B$, doctors with $r\in \left[{r}_{l}\left(t\right), {r}_{h}(t)\right]$ use the diagnostic test. If $t>g\left(1-g\right)\left(1-\lambda \right)B$, no doctor uses the diagnostic test.

**Fig. 2:**
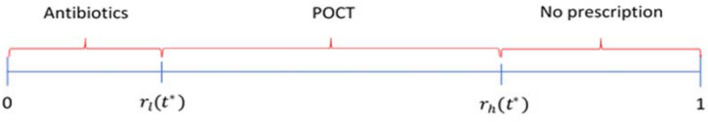
Use of POCT

The result stated in proposition 2 is quite intuitive. When the price of the diagnostic test is relatively high, all doctors prefer not to use it. When the price of the diagnostic test is relatively low, doctors with intermediate values of $r$ decide to use the diagnostic test. Doctors who are less concerned about resistance (those with relatively low values of $r$) do not use the diagnostic test and prescribe antibiotics to all patients. Doctors who are more concerned about resistance (those with relatively high values of $r$) do not use the diagnostic test either and leave patients without treatment. Notice that ${r}_{l}\left(t\right)\le {r}_{h}(t)$ for $t\le g\left(1-g\right)\left(1-\lambda \right)B$ (Fig. 2).

## The determination of the price of the diagnostic test

It follows from proposition 2 that the firm chooses a price for the diagnostic test $t\le g\left(1-g\right)\left(1-\lambda \right)B$. Doctors with $r\in \left[{r}_{l}\left(t\right), {r}_{h}(t)\right]$ use the test and the firm’s profits are $\Pi \left(t\right)=$ $t\left[F\left({r}_{h}\left(t\right)\right)-F\left({r}_{l}\left(t\right)\right)\right]$. Thus, the firm solves the following problem:

$$\underset{t}{\mathrm{max}}t[F\left({r}_{h}\left(t\right)\right)-F\left({r}_{l}\left(t\right)\right)]$$

$$s.t\quad t \in \left[ {0, g\left( {1 - g} \right)\left( {1 - \lambda } \right)B} \right]$$

The price ${t}^{*}$ of the diagnostic test that maximizes the firm’s profits satisfies the first order condition:

$$F\left({r}_{h}\left({t}^{*} \right)\right)-F\left({r}_{l}\left({t}^{*} \right)\right)+t\left[\frac{d{r}_{h}\left(t\right)}{dt}f\left({r}_{h}\left({t}^{*} \right)\right)-\frac{d{r}_{l}\left(t\right)}{dt}f\left({r}_{l}\left({t}^{*} \right)\right)\right]=0$$

As $\frac{d\Pi \left(t\right)}{dt}>0$ for $t=0$ and $\frac{d\Pi \left(t\right)}{dt}<0$ for $t= g\left(1-g\right)\left(1-\lambda \right)B$, it follows that ${t}^{*}$ $\in \left(0, g\left(1-g\right)\left(1-\lambda \right)B\right)$.^5^ Therefore, we have ${r}_{l}\left({t}^{*} \right)<{\overline{r}<r}_{h}\left({t}^{*}\right)$.

## Antibiotics prescriptions and health outcomes

In this section, we will first focus on the effect of the use of POCT on the number of prescriptions. As we know from proposition 2, doctors with $r\in \left[{r}_{l}\left({t}^{*}\right),{r}_{h}\left({t}^{*}\right)\right]$ use the diagnostic test. Doctors with $r\in [{r}_{l}\left({t}^{*}\right),\overline{r} ]$ gave antibiotics to all patients when they prescribed empirically. Now, these doctors use the diagnostic test and prescribe antibiotics only to the patients identified by the test as responders. Therefore, the number of prescriptions decreases in$\left(1-g\right)[F\left(\overline{r}\right)-F({r}_{l}\left({t}^{*}\right)]$. On the other hand, doctors with $r\in (\overline{r}, {r}_{h}\left({t}^{*}\right)]$ did not give antibiotics when they prescribed empirically. Now, these doctors use the diagnostic test and prescribe antibiotics to the responders identified by the test. Therefore, the number of prescriptions increases in$g[F\left({r}_{h}\left({t}^{*}\right)\right)-F\left(\overline{r}\right)]$. The net effect in the number of prescriptions is given by:

$$g\left[F\left({r}_{h}\left({t}^{*}\right)\right)-F\left(\overline{r}\right)\right]-\left(1-g\right)[F\left(\overline{r}\right)-F\left({r}_{l}\left({t}^{*}\right)\right]=g\left[F\left({r}_{h}\left({t}^{*}\right)\right)-F\left({r}_{l}\left({t}^{*}\right)\right)\right]-[F\left(\overline{r}\right)-F({r}_{l}\left({t}^{*}\right)]$$

The use of POCT increases the number of prescriptions if and only if:

$$g>\frac{F\left(\overline{r}\right)-F({r}_{l}\left({t}^{*}\right)}{F\left({r}_{h}\left({t}^{*}\right)\right)-F\left({r}_{l}\left({t}^{*}\right)\right)}$$

The left-hand side of this expression is the proportion of patients who benefit when treated with antibiotics. The right-hand side of the expression is the proportion of doctors who use POCT and stop prescribing antibiotic to patients who do not benefit from them. If this condition is not satisfied, the number of prescriptions is reduced or does not change.

Thus, it follows from the analysis that it should not be taken for granted that the use of diagnostic testing always reduces the number of antibiotics prescriptions and future AMR.

### Proposition 3:

When doctors use POCT, the number of antibiotics prescriptions increases if and only if $g>\frac{F\left(\overline{r}\right)-F({r}_{l}\left({t}^{*}\right))}{F\left({r}_{h}\left({t}^{*}\right)\right)-F\left({r}_{l}\left({t}^{*}\right)\right)}$. Otherwise, the number of antibiotics prescriptions either decreases or does not change.

With regard to health outcomes, they do improve with the use of POCT. Patients who are attended by doctors with $r\in [\overline{r}, {r}_{h}\left({t}^{*}\right)]$ are administered the test, and those identified as responders get a health gain. Patients who are attended by doctors with $r\in [{r}_{l}\left({t}^{*}\right),\overline{r}]$ are administered the test, but the responders do not get any health gain as they are given the same treatment as in the case of empiric prescription. A health gain is achieved with lower costs when the number of prescriptions is reduced. On the other hand, we have a health gain with higher costs when the number of prescriptions increases. Antibiotics are given to the patients who need them although both prescriptions and the negative effect on $AMR$ increase. When patients recover without treatment, although with lower health benefits, it may be better to not use POCT. In this situation, there would be a health loss but less antibiotics prescriptions.

## Discussion and conclusions

In this article, we have studied whether the use of POCT to guide treatment decisions for infectious diseases reduces antibiotics prescriptions. Most of the literature on the efficiency of the use of POCT is empirical. The economic evaluations performed to assess the cost-effectiveness of POCT (see Van der Pol et al. [8] for a systematic review of the use of POCT for respiratory tract infections) find POCT to be cost-effective only in some countries (e.g. the Netherlands) while POCT was dominated by usual care in others (e.g. Spain) [9, 10]. We have carried out a stylized theoretic economic model to analyse the effects of diagnostic testing in antibiotics prescription and health outcomes when doctors show concern for AMR. It is widely accepted that the use of diagnostic testing to guide antibiotics prescription in community care settings reduces the number of antibiotics prescriptions. Contrarily to this view, our model shows that we should be very careful with recommending the use of POCT to reduce antibiotics prescriptions as we may end up with the opposite result. We have found that the number of antibiotics prescriptions is not always lower. This result depends on the distribution of the doctors’ concern for AMR. There will be a proportion of doctors who use POCT and thus prescribe antibiotics while others doctors change their prescribing behaviour after using POCT and reduce antibiotics prescriptions. The number of antibiotics prescriptions does not change if the distribution is uniform. Nevertheless, we consider this distribution to be unlikely in the real world, so we should expect the number of antibiotics prescriptions either to be reduced or increased. The message our analysis tries to convey is that we should be very careful when we assert that POCT reduces antibiotics prescriptions as there are situations in which the contrary effect is produced. Our analysis also shows that the use of POCT improves health outcomes.

The theoretical economic literature on this issue is scarce. To the best of our knowledge, only Antoñanzas et al. [11] present a theoretic model to study the use of POCT to treat infectious diseases and its impact on antibiotics prescription, although their setting is different to ours. In their model, doctors differ in their degree of uncertainty when prescribing due to personal characteristics and experience. They find that the use of POCT reduces the number of antibiotic prescriptions. As usually happens with theoretic economic models dealing with the same research topic, the findings depend on the particular elements the authors use to define and characterize the situation they analyse.

Our static model presents some limitations. We have assumed the costs of antimicrobial resistance to be exogenous to the model. As these costs depend on current and future antibiotics prescriptions, perhaps a dynamic approach would have been a better option to model the effects of POCT on antibiotic prescription. This type of model is beyond the scope of our current study. We have assumed a binary decision for treatments: either all patients are given antibiotics or left untreated. Thus, we have implicitly assumed that some patients had a viral infection that did not require antibiotic treatment, although it is used in some cases for viral infections [12]. Instead, we could have assumed that these patients are given a treatment when they are identified by POCT. In that case, the decision to use POCT would have been affected as its cost would be higher, modifying the number of doctors willing to use POCT. We believe that this extension would not qualitatively change the results, as we would have the same trade-off between doctors who now prescribe antibiotics and doctors who give up prescribing them. Inappropriate antibiotics prescribing may also occur when antibiotics have no effect on the pathogen. In our model, we have assumed that antibiotics prescribing is appropriate only for a proportion of patients $g$, without specifying why it is inappropriate for the remaining patients. We have implicitly assumed that either the infection could be of viral origin, or antibiotics are not effective against the pathogen causing the infection, or patients recover without being treated. The use of POCT reduces antibiotics prescriptions for viral infections (in our model, some patients who do not need treatment and are identified by the POCT), although it does not have any impact on reducing prescriptions when antibiotics are inappropriate to treat the bacterial infection due to AMR. In the model, the POCT does not indicate the appropriate antibiotic treatment. We are also aware that inappropriate antibiotic treatments increase AMR. Future research should consider this issue.

Finally, we have assumed that there is only one firm that sells the POCT. In the real world, there is competition in the market for diagnostic testing devices, and firms differ in the quality of their products (sensitivity and specificity). We have assumed for the sake of simplicity that POCT perfectly identifies patients who would benefit from treatment with antibiotics. The introduction of POCT differentiation and competition in the model would make the analysis more realistic. We hope to explore this extension in further research.

## Footnotes

1 Alternatively, we could have considered that doctors are able to identify a proportion of patients $\alpha$ for whom antibiotics are effective and a proportion of patients $\beta$ who do not require antibiotics, but there is a proportion $1-\alpha -\beta$ of patients for whom doctors are uncertain about the right treatment, and believe that antibiotics are effective for only a proportion $g$, although doctors are unable to identify such patients. POCT eliminates the uncertainty and identifies these patients. Thus, doctors may prescribe effectively antibiotics to a proportion of patients ${\upalpha }$ (patients with a bacterial infection who are prescribed the appropriate antibiotic) and not prescribe antibiotics to a proportion of patients $\upbeta$ (for example, doctors consider that these patients do not require antibiotics treatment as they suffer from a viral infection or can be cured without treatment). The approach we follow simplifies the notation without affecting the qualitative results.

2 The cost of antimicrobial resistance associated with the consumption of one unit of antibiotics ranged, in the US, from $$\$0.1$$ for carbapenems to $$\$0.6$$ for quinolones, cephalosporins and broad-spectrum penicillin. The costs were adjusted to 2016 US$ [7].

3 Hereafter, we will refer to a doctor with AMR concern $r$ as doctor $r$.

4 If $g\left(1-\lambda \right)B-p\le 0$, no doctor prescribes antibiotics as $\overline{r}\le 0$. The use of POCT obviously increases the number of prescriptions. If $g\left(1-\lambda \right)B-p\ge c$, all doctors prescribe antibiotics as $\overline{r}\ge 1$. The use of a diagnostic test obviously reduces the number of prescriptions. The analysis of these extreme situations is available upon request.

5 Alternatively, as $\Pi \left(0\right)=\Pi \left(g\left(1-g\right)\left(1-\lambda \right)B\right)=0$, the solution is interior.
